# Post-discharge telephonic follow-up of pediatric patients affected by SARS-CoV2 infection in a single Italianpediatric COVID center: a safe and feasible way to monitor children after hospitalization

**DOI:** 10.1186/s13052-021-01065-w

**Published:** 2021-06-02

**Authors:** Vitangelo Clemente, Costanza Tripiciano, Patrizio Moras, Daniele Deriu, Martina Di Giuseppe, Anna Lucia Piscitelli, Michela Cammerata, Maria Antonietta De Ioris, Francesca Ippolita Calò Carducci, Stefania Bernardi, Andrea Campana, Patrizia D’Argenio, Paolo Rossi

**Affiliations:** 1 Academic Department of Pediatrics (DPUO), Unit of Perinatal Infection and Congenital Infectious Diseases, Bambino Gesù IRCCS Pediatric Hospital, Rome, Italy; 2 Pediatric Academic Department, University of Rome Tor Vergata, Rome, Italy; 3 Pediatric Department, Bambino Gesù IRCCS Pediatric Hospital, Rome, Italy

**Keywords:** SARS-CoV-2, Follow-up, Pediatric, Telephonic

## Abstract

**Background:**

SARS-CoV-2 infection in children is often non severe and in the majority of cases does not require long term hospitalization, nevertheless it is burdened with social issues and managing difficulties.

To our knowledge there is no literature on telephonic follow up in pediatric patients with positive PCR for SARS-CoV-2 on rhino-pharyngeal swab after discharge. The aim of the study is to describe our experience in a telephonic follow up which can allow early and safe discharge from hospital while keeping the patients under close clinical monitoring.

**Materials and methods:**

Sixty-five children were admitted for SARS-CoV-2 infection at Bambino Gesù Pediatric Hospital COVID Center from 16th March to 3rd July. We monitored through a telephonic follow-up, using a specific survey, the patients discharged still presenting a positive PCR for SARS-CoV-2. We checked if any symptoms occurred at home until recovery, defined as two consecutive negative PCR for SARS-CoV-2 on rhino-pharyngeal swabs.

**Results:**

During the follow up 7 patients had mild and self-limited symptoms related to SARS-CoV-2 infection, while 2 patients were re-hospitalized. One patient had Multisystem Inflammatory Syndrome in Children (MIS-C), the other patient had an increase in troponin and D-dimers.

We also monitored the average time of viral shedding, resulting in a median duration of 28 days.

**Conclusion:**

Our experience describes the daily telephonic follow up as safe in pediatric patients discharged with positive PCR. As a matter of fact it could avoid long term hospitalization and allow to promptly re-hospitalize children with major complications such as MIS-C.

## Introduction

In early January 2020 a novel type of Coronavirus (CoV) was identified in a patient affected by pneumonia of unknown origin [1]. The novel coronavirus (2019-nCoV) [2–4] rapidly spread worldwide, forcing the World Health Organization (WHO) to declare the outbreak as a pandemic on 11th March. The disease was named COVID-19 (Coronavirus Disease 2019) [5, 6] and the virus SARS-CoV-2 by the International Committee on Virus Taxonomy.

Italy was among the first countries in the world to be affected by the COVID-19 outbreak, with 1.2% of all patients represented by children [7–13]. According to the Italian Istituto Superiore di Sanità (ISS), the estimated overall lethality in Italian patients was 3,4%. Specifically, in pediatric setting the lethality was 0.01% between the age of 0 and 19 years [14].

Molecular-based approaches are the first line methods to confirm suspected cases of SARS-CoV-2 infection. Nucleic acid testing is the main technique for laboratory diagnosis. Other methods such as virus antigen or serological antibody testing are also valuable assays with a short turnaround time [15]. The sensitivity and specificity of rhino-pharyngeal swabs for the diagnosis of COVID-19 is not well known. It seems to be very specific, but moderately sensible (with previously published reports citing 63–78%), so a negative test does not rule out with confidence the possibility of a SARS-CoV-2 infection. The Real Time-PCR analysis of bronchoalveolar lavage fluid is the most accurate, but it could be performed easily only in patients admitted to a pediatric intensive care unit. The nasal swabs have a higher sensitivity than the pharyngeal [16].

Based on the global interest and concern about COVID-19 several studies have reviewed symptoms and characteristics of adults with SARS-CoV-2 infection [17]. Given the lower incidence in pediatric patients, there are fewer studies in this cohort [18–22].

Children mainly acquire SARS-CoV-2 infection from their family members but seem to experience a less severe form of COVID-19 disease than adults. Most of times they are asymptomatic or experience mild symptoms [23]. Frequent clinical manifestations include fever, dry cough and fatigue accompanied by other upper respiratory symptoms, such as nasal congestion and runny nose, pneumonia, dyspnea, headache and arthralgia. Moreover, the main gastrointestinal symptoms are nausea, vomiting and diarrhea [23]. An important complication of the SARS-CoV-2 infection is the Multisystem Inflammatory Syndrome in Children (MIS-C), whose clinical presentation includes fever and involvement of two or more organs, associated with laboratory evidence of inflammation. MIS-C has some similarities with Kawasaki Disease and secondary hemophagocytic lymphohistiocytosis macrophage activation syndrome [24–26].

A previous work has described the potential role of telemedicine in response to public health emergencies [27]. Telemedicine has shown to be helpful in previous outbreaks caused by SARS-CoV (severe acute respiratory syndrome–associated coronavirus) MERS-CoV (Middle East respiratory syndrome coronavirus), Ebola and Zika viruses. Ohanessian R in 2015 proposed a model for telemedicine during public health emergencies, based on tele-expertise, remote patient monitoring of contact cases, and teleconsultation for triage and isolated cases [28]. However in most countries telemedicine is not well integrated with national health care systems. For example in Italy telemedicine was not implemented by health authorities during the first phase of the SARS-CoV-2 epidemic [29].

The aim of this study is to describe our experience with telemedicine, through a telephonic follow-up model, which can allow an early and safe discharge of children with a positive PCR for SARS-CoV-2, while keeping them under close clinical monitoring.

## Materials and methods

Sixty-five children aged between 10 days and 210 months were admitted for SARS-CoV-2 infection, confirmed by positive PCR on rhino-pharyngeal swab, at Bambino Gesù Pediatric Hospital COVID Center, from 16th March to 3rd July. Among these, 19 patients were discharged after remission of symptoms, still presenting a positive PCR on rhino-pharyngeal swab. We monitored through a telephonic follow-up all these 19 patients.

The follow up was performed during working hours by seven residents of the Bambino Gesù Children Hospital, through 2 calls per day to the patients’ parents. The calls had a variable length (from 2 to 10 min), influenced by numerous variables (e.g., patient conditions, parents’ questions, etc.). A specific survey was used (Table 1), in order to check if SARS-CoV-2 - related symptoms appeared [23], if any medication was administrated to the patient and if the rhino-pharyngeal swab was performed. In the case of the appearance of new symptoms, if necessary, the resident gave some management advices (e.g., administration of paracetamol in case of fever). The residents communicated the relevant information obtained from the phone calls to a physician of the Bambino Gesù Children Hospital COVID center.

**Table 1:** Post-discharge telephonic survey

The resident introduces himself (Full Name, Qualification)	
Which have been the general clinical conditions of the child in the last 12 h?	
Has he had hyperpyrexia in the last 12 h? If yes, which was his temperature?	
Has he had breath difficulties in the last 12 h?	
Has he coughed in the last 12 h?	
Has he had sore throat in the last 12 h?	
Has he had rhinitis in the last 12 h?	
Has he had diarrhea or abdominal pain in the last 12 h?	
Has he had a headache in the last 12 h?	
Has he had arthralgia in the last 12 h?	
Has he manifested other symptoms in the last 12 h?	
Has he had another rhino-pharyngeal swab done? If so, which was the result?	

The telephonic follow up was taken forward until two consecutive negative PCR for SARS-CoV-2 were achieved. Two consecutive PCR 24 h apart were necessary to increase the test senitivity. All the rhino-pharyngeal swabs were performed weekly by the local health authority at patients’ home.

We tabulated the records through a Microsoft Excel Software, and we used descriptive statistics for the analysis of the data.

## Results

Our cohort consisted of 19 children aged between 8 and 188 months (Table 2). Among these, 13 patients were male and 6 female. 3 of our patients presented comorbidities: 1 patient was affected by Angelmann syndrome, 1 patient had Congenital Arthrogryposis, 1 patient had Kikuchi Syndrome.

**Table 2:** Patients’ demographic and clinical features

Patient	Gender	Age (years)	Comorbidities	Symptoms at the onset	WBC 103/μL	Neutrophils 103/μL	Lymphocytes 103/μL	CRP mg/dl	Radiographic Findings: 0 = normal; 1 = interstitial lung involvement; 2 = pulmonary consolidation	Days of hospitalization	Therapy during the hospitalization	Symptoms Relapse	Days of Follow up
1	F	3,8	NO	Fever, febrile seizure	8.6	4.15	3	0.14	0	5	none	NO	48
2	M	0,7	NO	Fever, cough	13	7.84	3.12	0.49	0	8	none	NO	25
3	M	6,7	Congenital Arthrogryposis, Cerebellar hypoplasia, Neurodevelopmental delay	Fever, cough, acute respiratory failure	5,75	4.69	0.54	0.24	1	14	cefriaxone, clarithromycin, oxygen therapy	NO	14
4	M	13,7	NO	Fever, cough	5.44	2.32	2.26	0.66	0	9	none	Fever, Vomiting, Loss of appetite	53
5	F	15,7	Immune thrombocytopenia	Fever, dyspnea	2.79	1.2	1.34	0.37	1 + 2	6	hydroxychloroquine, azithromycin	Low-grade fever	34
6	F	9,7	Angelman Syndrome	Fever, seizures	6.35	3.81	1.38	0.25	1	9	none	Fever	19
7	M	10,9	NO	Fever, cough, rhinitis, conjunctivitis	5.62	1.34	2.81	0.03	0	6	none	Rash	19
8	M	11,5	Hypoacusia	Fever, headache, arthralgia, diarrhea	4.9	1.84	2.24	0.03	0	8	cefixime	Pharyngodynia, Conjunctivitis	17
9	M	10	Recurrent otitis media	Fever, headache, arthralgia, diarrhea	4.4	1.79	1.86	0.03	0	8	none	NO	16
10	M	8,5	NO	No	4.21	1.89	1.87	0.04	0	7	none	Abdominal pain, Headache, Cold sores	24
11	M	6,8	NO	Fever, myalgia	5.64	1.58	3.15	0.07	0	7	none	NO	30
12	M	6,8	NO	Fever	4.43	1	3.01	0.1	0	6	none	Myalgia of the lower limbs	11
13	F	15,3	NO	Fever, cough, anosmia	5.48	2.14	2.1	0.2	1	10	hydroxychloroquine, azithromycin	NO	25
14	F	2,3	NO	Cough	11.44	2.44	7.36	0.06	0	14	none	NO	15
15	M	9,6	NO	No	4.36	1.63	1.67	0.06	0	6	none	NO	13
16	M	0,7	NO	Diarrhea	5,93	0.88	3.88	0.03	0	6	none	Rash	28
17	M	13,2	Kikuchi Disease, Hemophagocytic Lymphohistiocystosis	Fever, cough	5.6	3.68	1.04	0.35	0	23	none	NO	5
18	F	1,3	NO	Fever, cough	8.450	3.92	3.36	0.06	1 + 2	12	hydroxychloroquine, azithromycin betamethasone	NO	26
19	M	5,9	NO	No	5.59	2.3	2.66	0.06	0	9	nessuna	NO	25

**Fig. 1:**
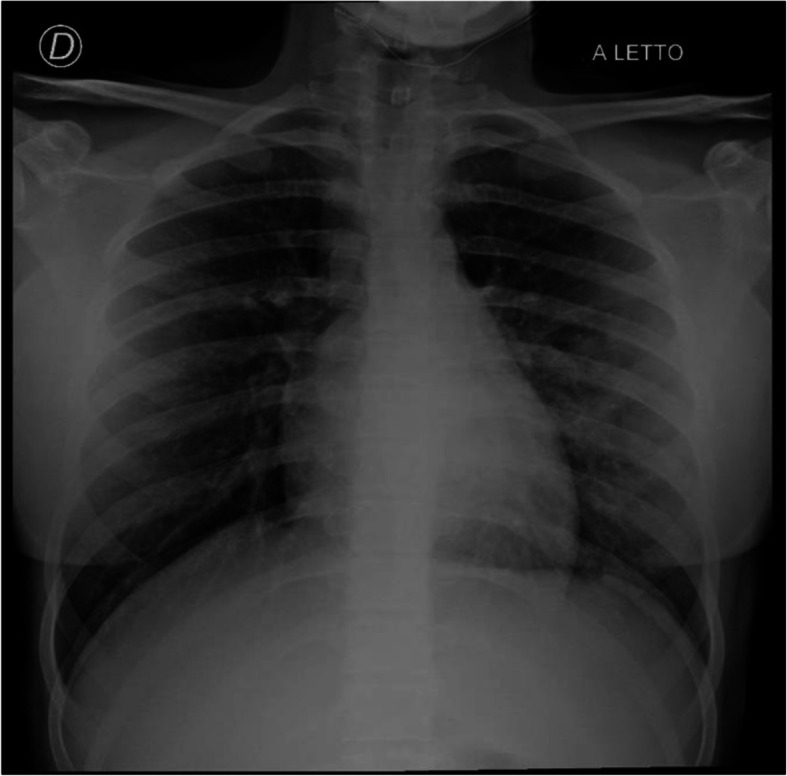
Patient’s chest X-ray: Interstitial involvement marked in hilar and basal left regions

**Fig. 2:**
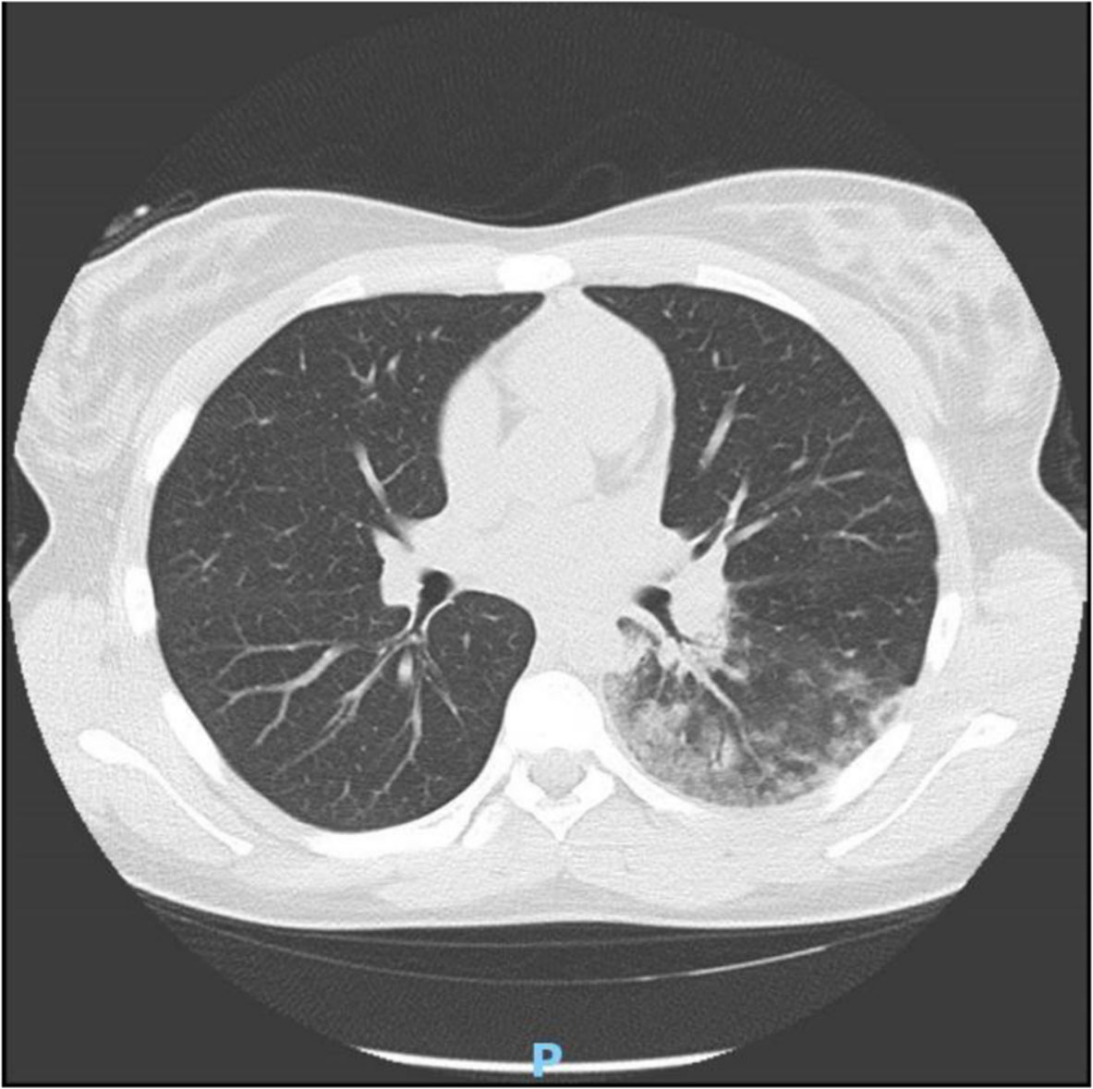
Patient’s lung CT: Multiple round and oval ground-glass opacities in the left lung

The most common symptoms were fever and cough, interstitial lung involvement on chest radiography was observed in 5 patients (Figs. 1 and 2). At admission, one patient had neutropenia, one patient had lymphopenia. All patients had normal levels of C reactive protein. Five of the 19 patients were treated with antibiotics during hospitalization, 1 patient required oxygen therapy. At discharge all patients were asymptomatic.

The average length of hospitalization of our cohort was approximately 10 days, with a range between 5 to 12 days. The time of hospitalization was in several cases influenced by non-clinical factors, including familiar and social issues; for example a domicile not adequate for isolation or difficulties of the local health authority in performing rhino-pharyngeal swab to the patients’ home.

All the 19 patients were contacted during the follow-up period and 7 of these patients presented new onset symptoms, described in literature as expression of SARS-CoV-2 infection [23], such as sore throat, conjunctivitis, cough, abdominal pain, fever, headache, myalgia and facial rash (1 for each child). Most of the symptoms were mild and healed in a few days*.*

Two patients were re-hospitalized for complications related to SARS-CoV-2 infection: one patient presented with MIS-C, the other patient had an increase in D-dimers and troponins 28 days after discharge. Both complications were already described in patients with SARS-CoV-2 infection [26, 30].

We also monitored the amount of time the PCR for SARS-CoV-2 took to become negative: the median of viral shedding was 28 days, with values between 15 and 59 days.

## Discussion

There is little experience on follow-up in children with SARS-CoV-2 infection [31, 32]. Furthermore, to our knowledge there is no literature on the telephonic follow-up in pediatric patients with positive PCR on rhino-pharyngeal swab after discharge.

In our cohort 2 patients were re-hospitalized for the appearance of complications due to SARS-CoV-2 infections.

We compared the average of viral shedding with the data collected by De Ioris et al. and Hongmei Xu et al., and we observed that in our cohort the PCR for SARS-CoV-2 took a longer time to become negative [33, 34]. Although SARS-CoV-2 infection in the pediatric population is most of the time non severe and often asymptomatic, children’s infection is burdened with social issues and management difficulties, due to the need of familial or parental assistance during hospitalization. Early and safe hospital discharge is therefore essential in pediatric patients affected by COVID-19. A telephonic post-discharge follow-up could help to achieve a briefer hospitalization and an early detection of potential complications of the SARS-CoV-2 infection. As a matter of fact we were able to detect at an early stage the patient who presented with MIS-C, which represents a severe complication related to SARS-CoV-2 infection in children, and whose prognosis is influenced by the promptness of treatment [35].

Telehealth could allow to perform a more frequent assess of patients’ clinical conditions rather than ambulatorial follow up and could enable a decrease in the number of access to health structures during pandemic phases. Moreover, parents could feel more supervised and reassured through the daily conversation with the doctors of the pediatric COVID center.

In addiction briefer hospitalizations would enable a greater receptivity of the hospital during pandemic phases and would allow to reduce hospitalization related costs as described by Peong Gang Park et al. [31]

The biggest drawback of our study is the limited number of patients. Further studies will be necessary to assess the appropriate timing of calls, based on patients’ age and comorbidities.

## Conclusion

Our experience highlights the importance of telephonic follow-up in attempting to reduce hospitalization time in SARS-CoV-2 infected patients still presenting a positive PCR on rhino-pharyngeal swab. Finally with this close follow up it would be possible to identify at an early stage late complications related to SARS-CoV-2 infection.

## Data Availability

Not applicable.

## Abbreviation

MIS-C: Multisystem Inflammatory Syndrome in Children
